# Lineage Switch Macrophages Can Present Antigen

**DOI:** 10.1155/1992/34586

**Published:** 1992

**Authors:** James D. Bretz, Shu-Chih Chen, Diane Redenius, Hsun-Lang Chang, Walter J. Esselman, Richard C. Schwartz

**Affiliations:** Department of Microbiology Michigan State University East Lansing Michigan 48824-1101 USA

**Keywords:** Lineage switch, macrophage, antigen presentation

## Abstract

Recent reports of “lineage switching” from a lymphoid to macrophage phenotype have left unresolved the question of whether such cells are functional macrophages or nonfunctional products of differentiation gone awry. This study demonstrates that several “macrophage-like” cell lines derived from v-Ha-*ras*-transformed pre-B cells have gained the capacity to effectively present antigen in MHC-restricted fashion. Using an assay involving the cocultivation of putative antigen-presenting cells with chicken ovalbumin (cOVA) and a cOVA-specific T-cell hybridoma, “lineage switch” cell lines were found to present antigen as effectively as macrophage-containing peritoneal exudates. Neither the original pre-B-cell precursors nor B-cell lymphomas derived from them present antigen. Thus, we have demonstrated that these “lineage switch” macrophages are capable of antigen presentation, a mature differentiated function. While gaining macrophage characteristics, these cells have also rearranged their kappa light-chain immunoglobulin locus, suggesting that macrophage differentiation and immunoglobulin rearrangement are not mutually exclusive processes. The existence of both lymphoid and myeloid characteristics in a cell fully capable of antigen presentation suggests greater plasticity in hematopoietic lineage commitment than conventionally thought to be the case.


*Developmental Immunology*, 1992, Vol. 2, pp. 249–261
Reprints available directly from the publisher
Photocopying permitted by license only

© 1992 Harwood Academic Publishers GmbH
Printed in the United Kingdom

### Lineage Switch Macrophages Can Present Antigen

JAMES D. BRETZ, SHU-CHIH CHEN, DIANE REDENIUS, HSUN-LANG CHANG, WALTER J. ESSELMAN, and RICHARD C. SCHWARTZ*

*Department of Microbiology, Michigan State University, East Lansing, Michigan 48824-1101*

Recent reports of "lineage switching" from a lymphoid to macrophage phenotype have left unresolved the question of whether such cells are functional macrophages or nonfunctional products of differentiation gone awry. This study demonstrates that several "macrophage-like" cell lines derived from v-Ha-*ras*-transformed pre-B cells have gained the capacity to effectively present antigen in MHC-restricted fashion. Using an assay involving the cocultivation of putative antigen-presenting cells with chicken ovalbumin (cOVA) and a cOVA-specific T-cell hybridoma, "lineage switch" cell lines were found to present antigen as effectively as macrophage-containing peritoneal exudates. Neither the original pre-B-cell precursors nor B-cell lymphomas derived from them present antigen. Thus, we have demonstrated that these "lineage switch" macrophages are capable of antigen presentation, a mature differentiated function.

While gaining macrophage characteristics, these cells have also rearranged their kappa light-chain immunoglobulin locus, suggesting that macrophage differentiation and immunoglobulin rearrangement are not mutually exclusive processes. The existence of both lymphoid and myeloid characteristics in a cell fully capable of antigen presentation suggests greater plasticity in hematopoietic lineage commitment than conventionally thought to be the case.

KEYWORDS: Lineage switch, macrophage, antigen presentation.

### INTRODUCTION

The concept that hematopoietic differentiation involves an early and irreversible lineage commitment is brought into question by numerous observations of leukemias and lymphomas that express myeloid or lymphoid markers outside their respective lineages. The coexpression of differentiation markers has been interpreted as being either an aberrant phenomenon caused by leukemogenesis (McCulloch, 1983) or a reflection of the normal but transient existence of bipotential progenitors in hematopoiesis (Greaves et al., 1986). In particular, the existence of a number of transformed cell lines with both lymphoid and macrophage characteristics has suggested a close relationship between these lineages. Murine macrophage cell lines have been derived from lymphoid tumors and from in vitro transformants induced either by murine leukemia viruses or chemical carcinogens (Boyd and Schrader, 1982; Holmes et al., 1986; Hanecak et al., 1989). Three groups have studied systems in which a transition from a lymphoid to a macrophage phenotype could be induced. Klinken et al. (1988) demonstrated that B lymphoid cells from transgenic mice that express c-*myc* using the immunoglobulin mu enhancer could be induced to take on macrophage-like characteristics when infected with a retrovirus expressing v-*raf*. Davidson et al. (1988) showed that a v-Ha-*ras*-transformed lymphoid cell line could be stimulated by lipopolysaccharides (LPS) to differentiate along either the lymphoid pathway into pre–B-like cells or along the myeloid pathway into macrophage-like cells. Recently, Borzillo et al. (1990) reported the CSF-1-dependent macrophage lineage transition of a pre-B-cell line expressing the human CSF-1 receptor.

The macrophage-like cell lines that have been derived from B lymphoid cells have been classified as macrophage on the basis of their morphology, expression of MAC-1, MAC-2, $\alpha$-naphthyl acetate esterase and lysozyme, and their

*Corresponding author.

ability to phagocytose latex beads. More functional assays for antigen presentation and tumoricidal activity that would establish whether these cells could act in vivo similarly to authentic macrophages have not been presented. In this paper, we demonstrate the ability of several macrophage cell lines derived from v-Ha-*ras*-transformed pre-B cells to present antigen to a T-helper cell hybridoma.

### RESULTS

A tumor consisting of adherent cells with a macrophage morphology was identified during our studies on the tumor progression of a pre-B lymphoid cell line expressing v-H-*ras* (Chen et al., 1991). This tumor, designated tumor 4, was derived from a clonal cell line, designated R2, that was generated by infection of fresh murine bone marrow with a mixture of a v-Ha-*ras*-expressing retrovirus and Moloney murine leukemia virus (MoMuLV) (Schwartz et al., 1986b). The R2 cell line was classified as being a pre-B cell on the basis of several criteria. It possessed a blast-cell morphology with a large nucleus and scant cytoplasm. It expressed the B lineage-specific marker, B220 (Coffman and Weissman, 1981). Though not expressing a detectable immunoglobulin mu chain, R2 showed a rearrangement in the DNA of that locus. The immunoglobulin kappa-chain locus was in a germline configuration.

### Tumor 4 is Derived from the R2 Cell Line

In order to ascertain whether we had identified a probable instance of lineage switching, it was necessary to demonstrate that tumor 4 was derived from R2. To that end, the sites of integration of the v-Ha-*ras*-expressing retrovirus and MoMuLV were compared between the tumor and the cell line. Southern hybridization analysis of EcoRI-digested DNA with a v-Ha-*ras* probe showed that tumor 4 contained the same 5.3-kb proviral integration fragment as R2 (Fig. 1A). This proviral integration fragment is defined by a 3' EcoRI site internal to the viral genome and a 5' EcoRI site peculiar to the site of integration. In addition to the 5.3-kb fragment, there is a 23-kb fragment representing the endogenous c-Ha-*ras* in all the DNAs. Southern hybridization analysis of BglII-digested DNA, using a probe for the ecotropic MuLV *env* gene, revealed similar MoMuLV integration fragments in R2 and tumor 4 (Fig. 1B). The MoMuLV genome possesses a BglII site within *env*, such that the foregoing hybridization would detect a fragment extending from that BglII site to a BglII site in the host-cell genome flanking the 3' terminus of the provirus. These data demonstrate that the putative macrophage tumor was derived from the pre-B-cell line.

### Tumor 4 Cells Possess Macrophage Characteristics

Tumor 4 was initially suspected to be a macrophage because of the large size of its cells and its adherent growth in cell culture. Microscopic examination of Wright-Giemsa-stained cells confirmed their large size and revealed the cells of tumor 4 (Fig. 2B) to have a much more extensive and granular cytoplasm than R2 (Fig. 2A). An immunoperoxidase detection procedure found tumor-4 cells to have retained some expression of B220, and to have gained expression of high levels of MAC-1 (data not shown). MAC-1 is generally considered to be a marker for cells of the myeloid lineage (Springer et al., 1979). Histo-

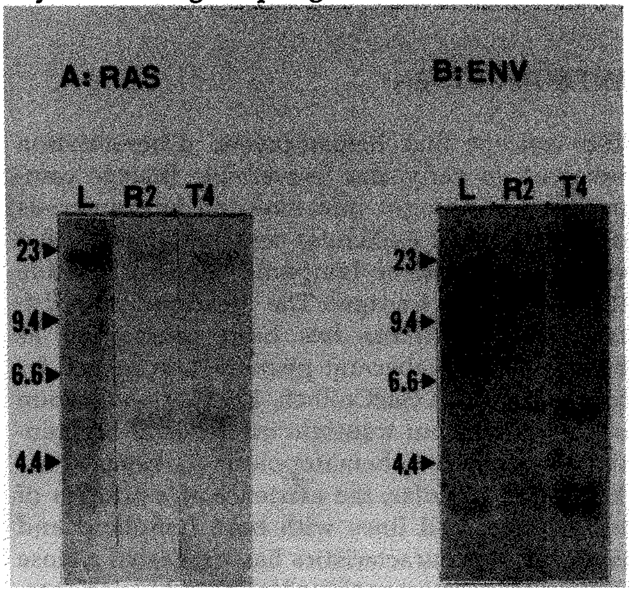

FIGURE 1. Viral intergrations. Southern blot analysis of DNAs from liver (L), R2, and tumor 4 (T4). (A) DNA was digested with EcoRI and 10 μg of each sample was electrophoresed through 0.8% agarose. The blot was probed for v-Ha-*ras*. (B) DNA was digested with BglII. The blot was probed for murine ectotropic *env* sequences. Size markers are the positions of an ethidium bromide-stained HindIII digest of bacteriophage λ and are denoted in kilobases.

chemical procedures revealed a high level of $\alpha$-naphthyl acetate esterase activity in tumor-4 cells, which is not found in R2 cells (data not shown). This is an enzyme activity generally associated with cells of the monocyte-macrophage lineage (Rogers et al., 1980). Tumor-4 cells (Fig. 2B) were positive for the nonspecific phagocytosis of latex beads, whereas R2 cells (Fig. 2A) were not. Nonspecific phagocytosis is another marker of the monocyte-macrophage lineage (Raschke et al., 1978). These data strongly suggest a macrophage phenotype for tumor-4 cells.

At late stages of myeloid differentiation, the levels of c-*myc* and c-*myb* mRNA decrease, whereas the level of c-*fms* mRNA increases (Gonda and Metcalf, 1984; Sheng-Ong et al., 1987). The levels of mRNA from these proto-oncogenes detected in tumor-4 cells were consistent with tumor 4 having advanced to a late stage of myeloid differentiation. Cytoplasmic poly A$^+$ RNAs of the parental R2 cell line, tumor 4, and six other tumors derived from R2 that had lymphoid characteristics were examined by

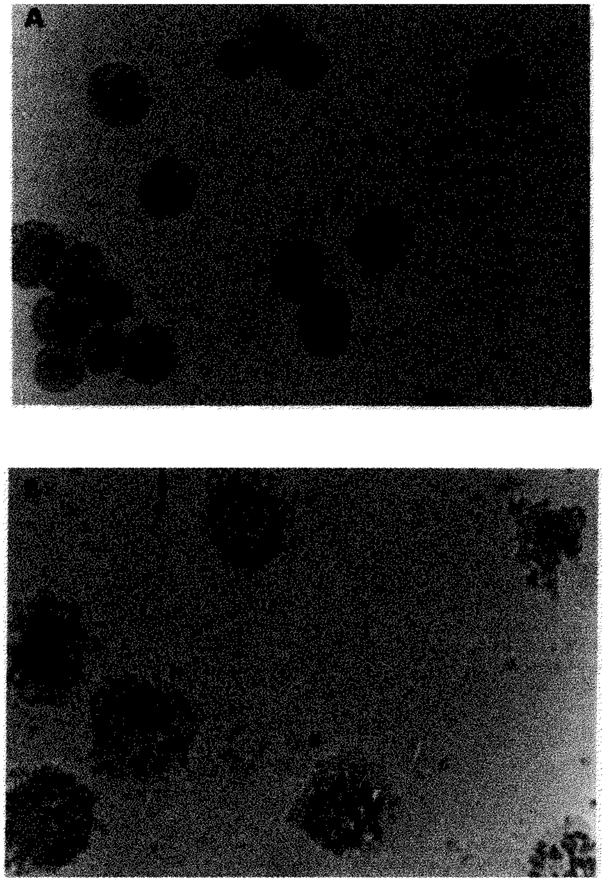

FIGURE 2. Nonspecific phagocytosis of latex beads. The cells were Wright-Giemsa stained and photographed at 200× magnification. (A) R2, and (B) tumor 4.

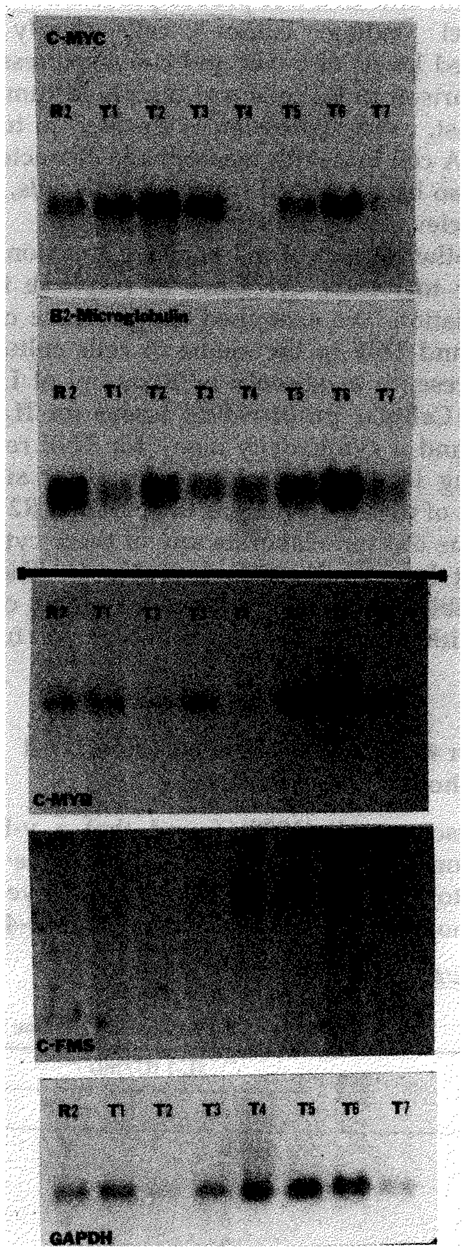

FIGURE 3. RNA analyses of c-*myc*, c-*myb*, and c-*fms*. Northern blot analyses were performed on polyA$^+$ RNA from R2 and seven tumors (T1–T7). Each sample of RNA was the polyA$^+$ fraction selected from 150 $\mu$g of total cytoplasmic RNA. One blot (upper panel) was probed successively for both c-*myc* and $\beta_2$-microglobulin, and the other blot (lower panel) was probed successively for c-*myb*, c-*fms*, and rGAPDH.

Northern hybridization analysis (Fig. 3). One blot was hybridized successively with c-*myc* and $\beta_2$-microglobulin probes. Another blot was hybridized successively with c-*myb*, c–*fms*, and rat glyceraldehyde-3-phosphate dehydrogenase (GAPDH) probes. Hybridization to the $\beta_2$-microglobulin and GAPDH probes provided a control for gel loading. Tumor-4 cells clearly show reduced levels of c-*myc* and c-*myb* expression in comparison to R2 cells and lymphoid tumors. In contrast, c-*fms* expression is elevated in tumor-4 cells. A cell line with macrophage characteristics has also been isolated from tumor-5 cells, which show elevated c-*fms* expression (Fig. 3).

Another aspect of macrophage function is the ability to release cytokines in response to LPS stimulation. We examined the presence of IL-1, IL-6, and TNF in the media of cells cultured in the presence or absence of 10 $\mu$g/ml of LPS for 24 hr. Cellular proliferation assays for IL-1 and IL-6, and a cytotoxicity assay for TNF revealed varying levels of cytokine release for six subclones of tumor 4, whereas the parental R2 pre-B-cell line did not elaborate any of these cytokines except low levels of IL-1 (Table 1). The LPS-inducible release of cytokines was again consistent with a macrophage phenotype for tumor-4 cells.

### Tumor 4 Cells Also Show Differentiated Lymphoid Characteristics

Davidson et al. (1988) found that a v-Ha-*ras*-transformed lymphoid cell line could be stimulated to differentiate along either the myeloid or lymphoid pathways. Because tumor-4 cells showed a variety of DNA rearrangements in the kappa light-chain locus (data not shown), it was of interest to determine whether the cells that had gone on to rearrange the kappa locus were the same cells that had progressed toward a macrophage phenotype or whether the tumor-4 cells were a mixed population of B cells and macrophages. To that end, tumor-4 cells were plated in soft agar medium and six subclones were recovered. Southern hybridization analysis of EcoRI-digested DNA isolated from the subclones showed that they all contained the same 5.3-kb v-Ha-*ras* proviral integration fragments as R2 and tumor-4 cells (data not shown; see Fig. 1A). The six subclones of tumor-4 possessed the same myeloid characteristics described before for the uncloned tumor, but varied in their pattern of kappa light-chain gene rearrangement. Southern hybridization analysis of BamHI-digested DNAs with a kappa probe revealed that subclones 3 and 5 possessed one germline and one rearranged kappa allele, and subclones 1, 2, 4, and 6 possessed rearrangements in both alleles (Fig. 4). All the subclones possessed a rearranged BamHI fragment of approximately 7 kb. Tumor 4 was apparently derived from an outgrowth of R2 that had undergone this rearrangement. Some subclones then proceeded to rearrange their other kappa allele. Clearly, tumor 4 contained cells that individually had differentiated along both the lymphoid and myeloid pathways.

Having observed kappa light-chain rearrangements in macrophage subclones of tumor 4, we next examined the status of immunoglobulin expression by Northern blot analysis. Kappa light-chain transcript could not be detected (data

TABLE 1
LPS-Induced Cytokine Release by Tumor-4 Macrophage Subclones[a]

| | IL-1 (U/ml) | | IL-6 (U/ml) | | TNF (U/ml) | |
|---|---|---|---|---|---|---|
| | –LPS | +LPS | –LPS | +LPS | –LPS | +LPS |
| R2 | 0 | 2 | 0 | 0 | 0 | 0 |
| T4.1 | 0 | 2 | 0 | 1 | 0 | 0 |
| T4.2 | 0 | 6 | 0 | 100 | 0 | 40 |
| T4.3 | 0 | 6 | 0 | 1500 | 0 | 85 |
| T4.4 | 0 | 19 | 0 | 39 | 0 | 34 |
| T4.5 | 1 | 4 | 0 | 0 | 0 | 0 |
| T4.6 | 0 | 4 | 0 | 35 | 0 | 0 |

[a]The capacity of cell lines to release the cytokines IL-1, IL-6, and tumor necrosis factor (TNF) was determined by assaying culture supernatants. For this purpose, cell lines were incubated for 24 h at $2.5\times10^5$ cells/ml with 10-$\mu$g/ml LPS in RPMI 1640 supplemented with 10% fetal calf serum and $5\times10^{-5}$ M 2-mercaptoethanol. Culture supernatants were collected, passed through a 0.2-micron filter, and stored at –70 °C until assayed. IL-1 activity was assayed by its ability to induce proliferation of D10.G4.1 cells in the presence of Concanavalin A as described by Ayala et al. (1990b). IL-6 activity was determined by its ability to induce the proliferation of the 7TD1 B-cell hybridoma as previously described by Hultner et al. (1989). TNF activity was assessed by its cytotoxicity to WEHI-164 clone 13 cells as previously described by Ayala et al. (1990a). The relative units of cytokine activity were determined by comparison of the activity of dilution series of experimental supernatants to the activities of dilution series of purified human IL-1 (Genzyme), recombinant human IL-6 (Amgen Corp.) or murine TNF-alpha (Amgen Corp.) standards.

not shown). A mu heavy-chain probe revealed a diverse range of RNAs in the macrophage subclones (Fig. 5) that correspond in size to 1.9-, 2.1-, 2.3-, and 2.9-kb transcripts reported to be initiated in the mu-switch region of myeloid cell lines (Kemp et al., 1980). The R2 pre-B-cell line possesses predominantly larger RNA species that include those that correspond in size to mature mu mRNAs of 2.4 and 2.7 kb. These species are diminished upon lineage switch. Comparison to a hybridization of the same blot with a probe for GAPDH (Fig. 5) shows the R2 RNA to be underloaded and thus the diminution of mu transcription in the macrophage is even more dramatic than apparent from casual inspection of the data. Apparently, the macrophage subclones of tumor 4 lose the capacity to transcribe functional mu mRNA, even though the rearrangement of the kappa locus suggests progress in lymphoid differentition.

Since CD45 isoforms have been reported to be lineage-specific (Ralph et al., 1987; Saga et al., 1987; Streuli et al., 1987), the expression of this surface marker was examined among the subclones of tumor 4. An immunoperoxidase-detection procedure detected B220, the B lymphoid isoform of CD45, in tumor-4 cells. The expression of CD45 was further examined among the tumor-4 subclones in order to determine the relative expression of the B220 isoform in comparison to the isoform that predominates in myeloid cells. Recently, Chang et al. (1989) described the use of a reverse transcription-polymerase chain reaction (RT-PCR) technique to determine the pattern of alternate exon use in CD45 expression of hematopoietic cells. They found that B lymphoid cell lines uniquely expressed a form of CD45 mRNA possessing three optional exons, whereas two myeloid cell lines (a macrophage and a mast cell) predominantly expressed a form lacking these exons. We utilized RT-PCR to examine CD45 expression among R2 and the subclones of tumor 4 (Fig. 6). All of the cell lines expressed multiple species of CD45 mRNA. Subclones 2, 3, 4, and 6 expressed a CD45 mRNA containing three optional exons, typical of B lymphoid cells, whereas subclones 1 and 5 predominantly expressed mRNA lacking these exons, typical of myeloid cells. R2 expressed the expected three exon B lymphoid isoform. Thus, the pattern of CD45 expression is heterogeneous among macrophage subclones of the same tumor.

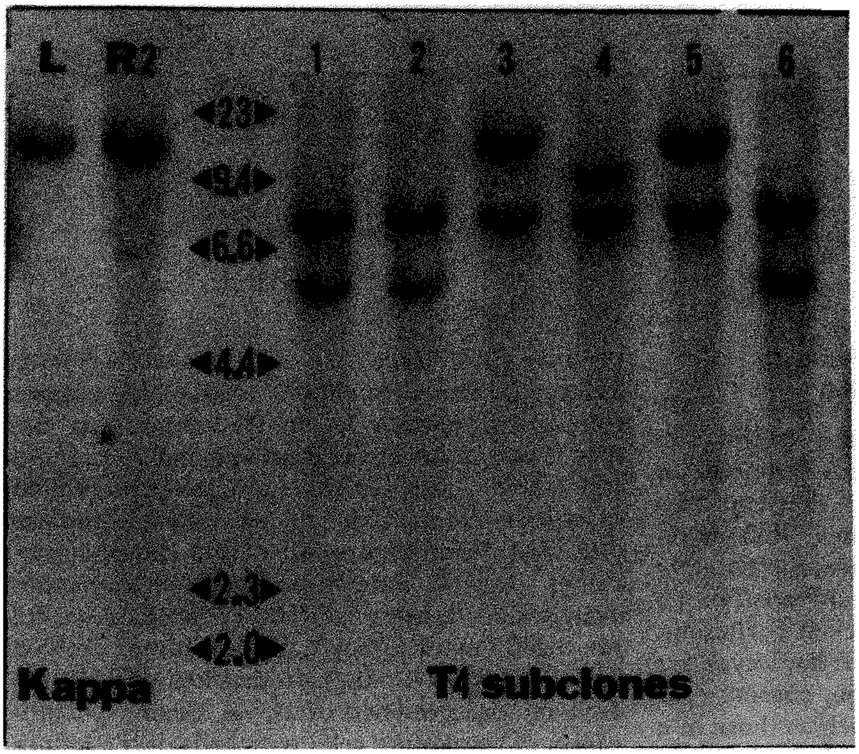

FIGURE 4. Kappa light-chain rearrangements. Southern blot analysis of DNAs from liver (L), R2, and six subclones of tumor 4 (1–6). DNA was digested with BamHI and 10 $\mu g$ of each sample was electrophoresed through a 0.8% agarose. The blot was probed for kappa light-chain constant region sequences. Size markers are the positions of an ethidium bromide-stained HindIII digest of bacteriophage $\lambda$ and are denoted in kilobases.

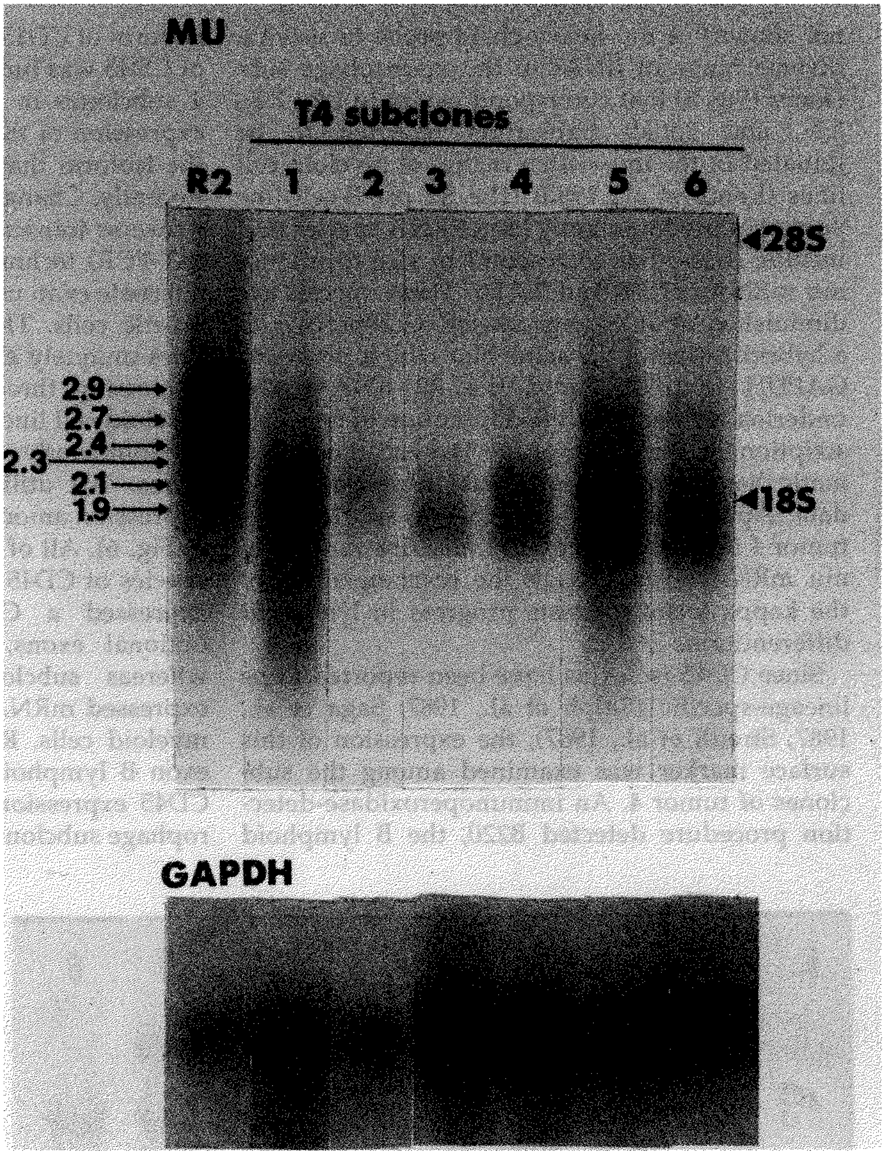

FIGURE 5. Expression of mu heavy-chain RNA. Northern blot analysis was performed by poly A+ RNA from R2 and six subclones of tumor 4 (1–6). Each sample of RNA was the poly A+ fraction selected from 100 μg of total cytoplasmic RNA. The blot was probed for mu heavy chain. The positions of ethidium bromide-stainëd rRNAs are noted on the right. The positions of mu RNA species are marked on the left and denoted in kilobases. The lower panel shows the same blot probed for GAPDH as a control for loading.

### The Subclones of Tumor 4 Can Function Effectively in Antigen Presentation

In order to test the ability of tumor-4 subclones to present antigen, an assay system required an antigen-specific T-helper cell line that could be stimulated to produce interleukin-2 (IL-2) upon presentation. For these experiments, the putative antigen-presenting cells were cocultivated with a T-cell hybridoma specific for chicken ovalbumin (cOVA) and restricted for I-A$^d$ (the haplotype for BALB/c), DO.11.10/54.4. In the presence of cOVA, authentic macrophages such as those in a peritoneal exudate stimulate the hybridoma to produce IL-2 (Fig. 7A). IL-2 production was assayed by the application of media supernatants from cocultivations to an IL-2-dependent cell line, CTLL-2. All of the macrophagelike tumor-4 subclones displayed antigen-presentation capacities comparable to peritoneal exudates (Fig. 7A). Furthermore, all of the tumor-4 subclones showed antigen-presentation capacities dramatically greater than either the parental R2 pre-B-cell line or tumor 1, a B-cell tumor derived from R2 (Fig. 7A). IL-2 production was dependent on the presence of cOVA during cocultivation of presenting cells with cells of the helper T-cell hybridoma. Supernatants produced in the

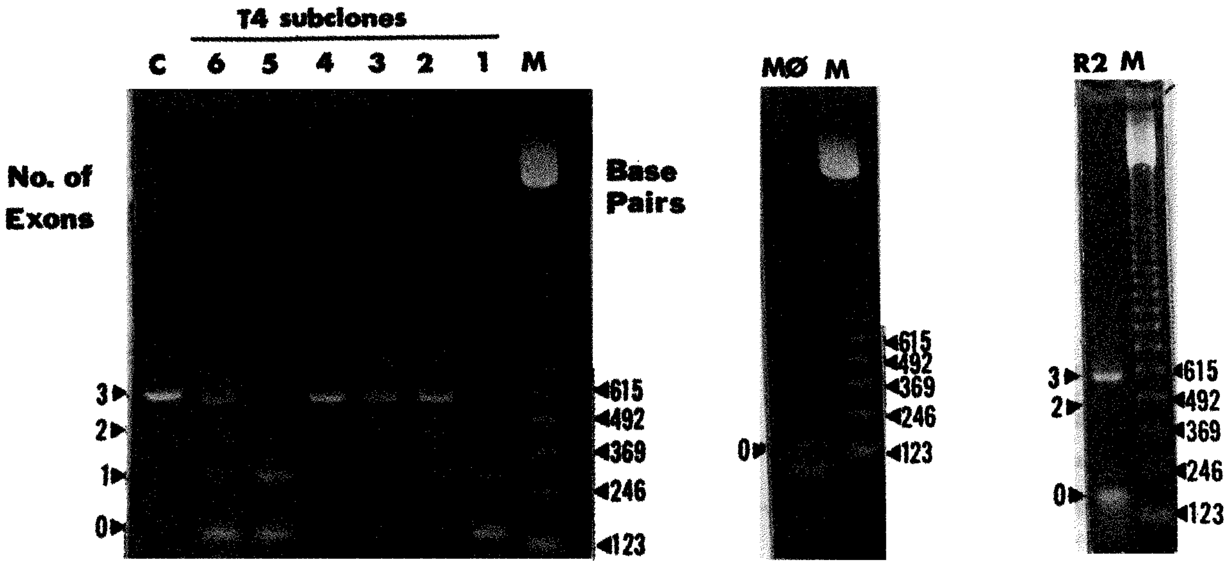

FIGURE 6. RT-PCR analysis of CD45. RT-PCR was performed on the polyA$^+$ RNAs of R2, the six subclones of tumor 4, and P388D1 (a myeloid control). The products were electrophoresed through 2% agarose and stained with ethidium bromide. (C) 3-exon plasmid control; (1–6) T4 subclones; (M) 123-bp ladder; (Mϕ). P388D. PCR products smaller than the 0 exon product may represent an RNA species lacking an additional exon (Chang and Esselman, unpublished results).

absence of cOVA were analyzed for all the cell lines and the values for IL-2 production were found to be near zero (data not shown). These control values were subtracted from those determined for supernatants produced in the presence of cOVA to generate the data presented in Figs. 7A, 7B, and 7C. IL-2 production was also dependent on the presence of T-cell hybridoma cells. Supernatants produced by incubations of putative presenting cells with cOVA in the absence of T-cell hybridoma cells had no detectable IL-2 (data not shown). The ability to present antigen was not stimulated by exposure to LPS for any of these cell lines (data not shown). A macrophage-like outgrowth from tumor 5 (also derived from R2) and the cells of a macrophagelike tumor derived from the pre-B-cell line R1 (9) showed levels of antigen presentation similar to those observed for the tumor-4 subclones (Fig. 7B). The ability to present antigen, therefore, may be a common phenomenon among v-Ha-*ras*-transformed B lymphoid cells that acquire macrophage-like characteristics.

Authentic antigenic presentation should be MHC-restricted, so all of the putative antigen-presenting cells were also cocultivated with a T-cell hybridoma specific for cOVA and restricted for I-A$^q$, 3Q023-24.4. As exemplified by subclone 4 of tumor 4 (T4.4) and the macrophage tumor derived from R1 (R1T), the antigen presentation observed is MHC-restricted (Fig. 7C).

### I-A Expression

Antigen presentation to T cells requires Ia expression and the observation of I-A$^d$-restricted presentation (Fig. 7C) indicates that these "lineage switch" macrophages express I-A$^d$. In order to assess whether the acquisition of presentation capacity correlated with acquisition of Ia expression, in particular I-A$^d$, we performed flow cytometry with FITC-conjugated antimouse I-A$^d$ on the macrophage cell lines and their pre-B-cell precursors. Although both R1 and R2 (pre-B cells) displayed no detectable I-A$^d$, the macrophage cell lines represented by R1T and T4.4 showed a low expression of I-A$^d$ (Fig. 8).

### DISCUSSION

This study demonstrates the capacity of several macrophage-like tumor cell lines derived from v-Ha-*ras*-transformed pre-B-cell lines to present antigen with MHC-restriction. This finding establishes that cells having undergone "lineage switching" can perform a function normally associated with a fully differentiated macrophage

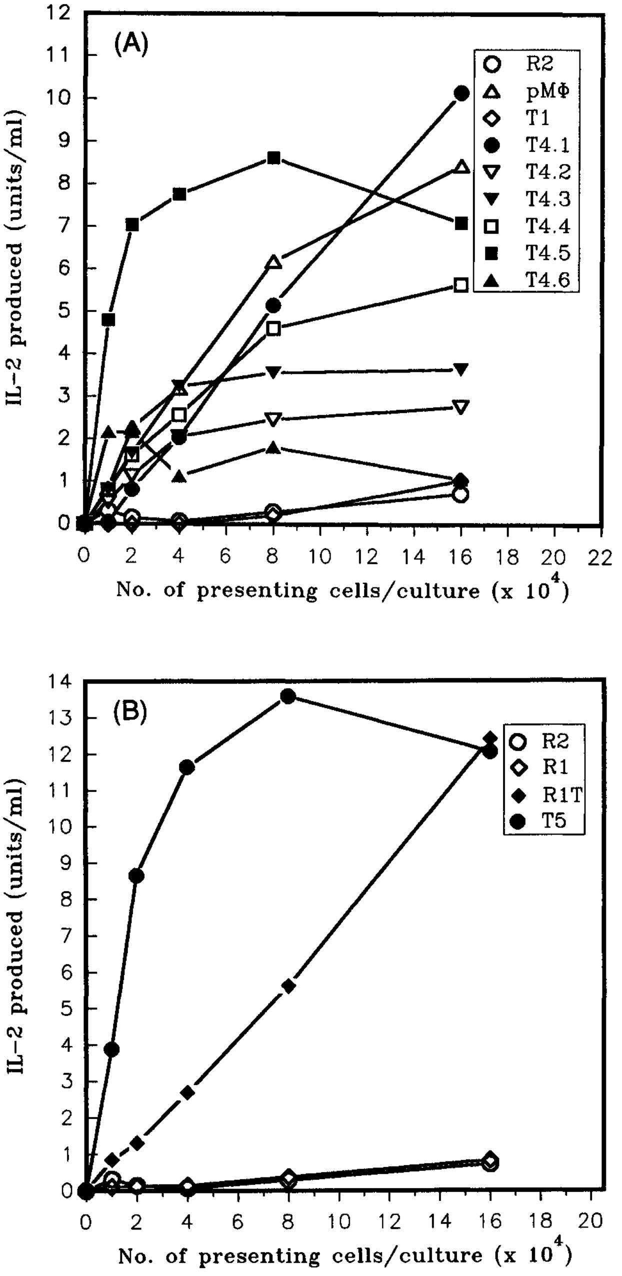

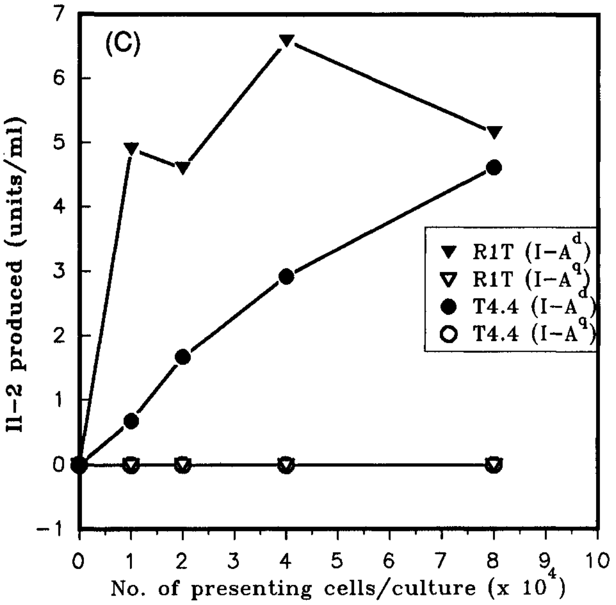

FIGURE 7

FIGURE 7. Antigen presentation. (A) Antigen-presentation assays were performed on R2, the tumor-4 subclones (4.1–4.6), tumor 1 (a B-cell tumor derived from R2), and peritoneal exudates (pM$\phi$). (B) Antigen-presentation assays were performed on R2, a macrophage outgrowth of tumor 5 (T5; derived from R2 in a different animal), R1 (another pre-B-cell line transformed by v-Ha-*ras*), and R1T (a macrophage tumor derived from R1). (C) Antigen-presentation assays were performed on a tumor-4 subclone (T4.4) and R1T with DO-11.10/54.4 (I-A$^d$-restricted) or 3Q023-24.4 (I-A$^q$-restricted). Each point represents an average value obtained from a dilution series for each presentation supernatant, testing the response of CTLL-2 cells to IL-2 in those supernatants. The results shown are representative of at least two experiments with each cell line.

or B cell. Although numerous examples exist of B lymphomas with the capacity to present antigen (Chesnut et al., 1982; Walker et al., 1982), neither the pre-B-cell precursors of the macrophage-like cell lines nor B-cell lymphoma cell lines derived from those precursors could present antigen. Thus, the capacity to present antigen appears to correlate with the differentiation of these cells along the macrophage lineage. Indeed, two cell lines with the most dramatic level of antigen presentation had lost expression of the B-cell isoform of CD45 and displayed a pattern of CD45 more typical of a myeloid cell (subclones 1 and 5, Fig. 6; T4.1 and T4.5, Fig. 7A). Perhaps, loss of the B-cell isoform of CD45 is indicative of further maturation along the myeloid lineage. It may be worthwhile to investigate the role of CD45 in macrophage function. The fact that similar antigen-presentation abilities were found in macrophage derivatives of two completely independent cell lines (R1 and R2) suggests the generality of this phenomenon.

Because it is well established that IL-1 along with antigen presentation is an important coactivator of T cells, it is surprising that the inducibility of cytokine release by LPS (Table 1) does not correlate with the effectiveness of antigen presentation by the T4 subclones (Fig. 7A).

Apparently the low levels of IL-1 that some of these macrophages are capable of elaborating is sufficient for T-cell activation. The observation that two of the best lines for antigen presentation (T4.1 and T4.5) have a weak response to LPS suggests that LPS-induced cytokine release may not be an adequate measure in itself for evaluating macrophage function.

The "lineage switch" macrophages reported here express a low level of Ia (Fig. 8). This is consistent with the previous report of Davidson et al. (1988). The precursor pre-B cells lack detectable Ia. Perhaps Ia expression is the critical property determining the capacity to present antigen among these cells. Certainly, Ia expression is necessary for antigen presentation, but its sufficiency for antigen presentation among the cell lines we have studied will require further experimentation.

The six macrophagelike subclones of tumor 4, while possessing a common rearranged kappa allele, displayed a variety of kappa light-chain gene rearrangements at their other kappa allele. Compared to their parental cell line, these cells have progressed along the B as well as the monocyte/macrophage lineage. The varying rearrangements of one kappa allele suggest rearrangement subsequent to macrophage conversion and that at least certain elements of lymphoid and macrophage differentiation programs are not mutually exclusive. The fact that the R2 cell line can also generate a lymphoma (T1, Fig. 7A) that expresses both mu and kappa chains (data not shown) demonstrates the potential of this cell line to differentiate quite far along either the lymphoid or macrophage pathways. The relationship between lymphoid and macrophage differentiation revealed in these cells differs somewhat from that seen in cases of "lineage switch" previously reported. Klinken et al. (1988) found "lineage switch" macrophages at both the pre-B- and B-cell stages of immunoglobulin rearrangement. However, they did not find macrophages that had progressed in their immunoglobulin rearrangement compared to their lymphoid cell precursors, as we have. Davidson et al. (1988), examining v-Ha-*ras*-transformants similar to those reported here, could induce those cells to differentiate into either lymphoid or macrophage cells upon exposure to LPS. The lymphoid derivatives they reported did not progress beyond the pre-B-cell stage, whereas we have identified an immunoglobulin-producing tumor derived from a pre-B-cell line that also gave rise to a macrophage tumor. Perhaps the more complex environment provided during tumor chal-

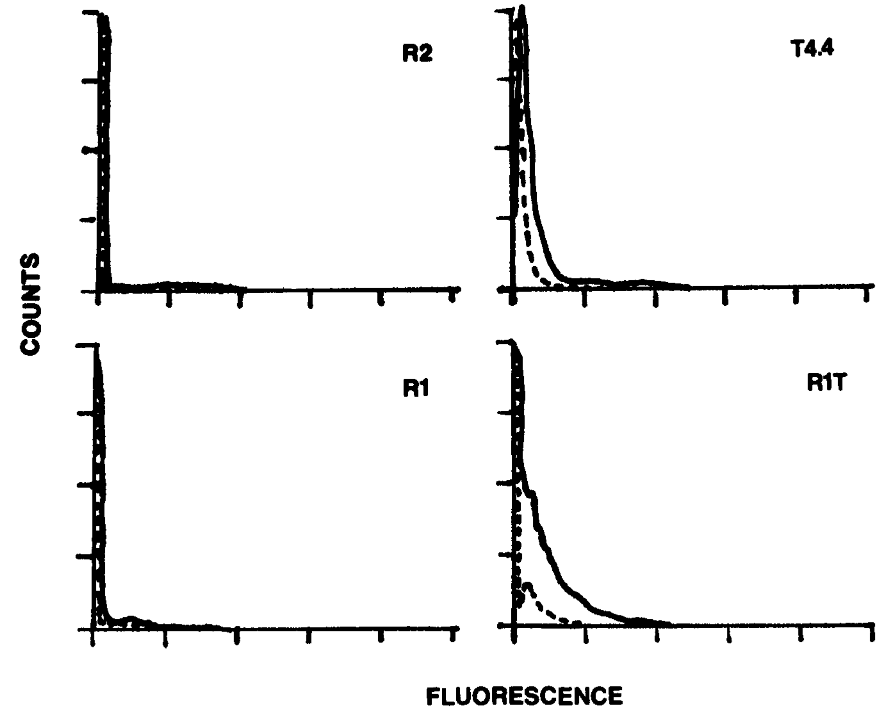

FIGURE 8. I-A$^d$ expression. Flow cytometry was performed on R1, R2, T4.4, and R1T after reactions with FITC-conjugated anti-I-A$^d$ (solid line) or, as a control, FITC-conjugated mouse $IgG_{2b}$,K (dashed line). For R1 and R2, the plots of experimental and control are virtually coincidental.

lenge allowed the cells described here to more fully develop along the lymphoid lineage when that pathway was selected. At any rate, the v-Ha-*ras*-transformed pre-B cells described here seem truly bipotential.

The ability of these cells that undergo an apparent "lineage switch" to perform a fully differentiated function presents the possibility that they may represent an unusual but normal subset of hematopoetic cells rather than an oddity induced by transformation. The existence of both lymphoid and macrophage characteristics in a cell fully capable of antigen presentation suggests greater plasticity in hematopoietic lineage commitment than conventionally thought to be the case.

### MATERIALS AND METHODS

### Cell Lines

R1 and R2 are v-Ha-*ras*-transformed murine pre-B-cell lines described in Schwartz et al. (1986b). Tumors derived from R1 and R2 were generated as described in Schwartz et al. (1986a, 1986b) in syngeneic BALB/c mice and in BALB/c athymic nude mice. Briefly, cells were washed twice in RPMI 1640 and were then resuspended in the same at $8\times10^6$ cells per ml. Five-week-old mice were injected intraperitoneally with 0.25 ml of the cellular suspension. Tumor 4, in particular, was isolated from an inguinal lymph node at 74 days postinjection. Tumor cell lines were readily produced from explanted tumors by dispersal and transfer to feeder cultures of adherent bone marrow cells (Whitlock et al., 1983). All of these cell lines were cultured over feeder cells in RPMI 1640 supplemented with 5% fetal calf serum and $5\times10^{-5}$ M 2-mercaptoethanol.

The subclones of tumor 4 were generated from single colonies grown in soft agar medium as described by Whitlock et al. (1983). The T-cell hybridoma, DO-11.10/54.4, was a generous gift of Drs. Philippa Marrack and John Kappler (University of Colorado, Denver) (White et al., 1983). This hybridoma is specific for chicken ovalbumin in the context of I-$A^d$ and cross reacts weakly with chicken ovalbumin in the context of I-$A^b$. 3Q023-24.4, another T-cell hybridoma, was also a gift of Drs. Marrack and Kappler. This hybridoma is specific for chicken ovalbumin in the context of either I-$A^q$ or I-E. CTLL-2 is a T-cell line responsive to IL-2 and was obtained from the ATCC. All of these cell line were cultured in RPMI 1640 supplemented with 10% fetal calf serum and $5\times10^{-5}$ M 2-mercaptoethanol in the absence of any feeder cells.

Peritoneal exudates containing macrophages were produced from BALB/c mice treated 1 week previously with a 0.5-ml intraperitoneal injection of pristane.

### Nucleic Acid Analysis

Cytoplasmic RNA was isolated from actively growing cells by a sodium dodecyl sulfate-urea procedure as described by Schwartz et al. (1981). Poly $A^+$ RNA was selected by oligo-dT cellulose chromatography (Aviv and Leder, 1975). RNA was denatured, electrophoresed in a formaldehyde-1% agarose gel (Rave et al., 1979), and transferred to Nytran (Schleicher and Schuell) (Thomas, 1980).

High molecular weight DNA was isolated from nuclei collected in the preceding RNA isolation procedure as described in Schwartz et al. (1986b). DNA was digested with restriction enzymes as noted in the figure legends, electrophoresed through 0.8% agarose, and transferred to Nytran (Southern, 1975).

Hybridization probes were prepared by nick translation (Rigby et al., 1979) through the incorporation of [$\alpha$-$^{32}$P] dATP (3000 Ci/mmol; ICN). The v-Ha-*ras* probe was the replicative form of phage M13mp10 containing a 0.46-kb EcoRI fragment corresponding to v-Ha-*ras* encoding sequences (Ellis et al., 1980). The *env* probe was a 0.8-kb BamHI fragment from the *env* region of Friend murine leukemia virus and is specific for the *env* sequences of murine ecotropic retroviruses (Silver and Kozak, 1986). The c-*myc* probe was the 4.7-kb genomic HindIII fragment of murine c-*myc* (Stanton et al., 1984). The murine c-*myb* probe was a cloned 2.4-kb cDNA (a generous gift of Dr. Timothy Bender, University of Virginia, Charlottesville). The *fms* probe was a cloned 2.7-kb ClaI-BamHI fragment of the McDonough strain of feline sarcoma virus (Donner et al., 1982). The murine $\beta_2$-microglobulin probe was a cloned 0.5-kb cDNA (Parnes et al., 1981). The rat glyceraldehyde-3-phosphate dehydrogenase (GAPDH) probe was a cloned 1.3-kb cDNA (Fort et al., 1985). The murine kappa light-chain probe was the replicative form of phage M13mp10 containing a genomic 0.48 kb HpaI-BglII fragment extending from a point about 50 base pairs within the 5' terminus of the kappa light-chain constant region gene to the poly A addition site (Seidman and Leder, 1978). The murine mu heavy-chain probe was a cloned cDNA ($\mu$12) that extends from $C_H2$ to the 3'-untranslated region of the secreted form of mu mRNA (Rogers et al., 1980). All hybridizations were performed under aqueous conditions in 5× SSC at 65 °C and washed to a stringency of 0.1× SSC at 65 °C.

### Reverse Transcription-Polymerase Chain Reaction (RT-PCR)

RT-PCR was performed according to the procedure of Chang et al. (1989, 1991) using poly $A^+$ RNA as substrate. The primers were a sense primer specific to exon 2 (GCCCTTCTGGACACAGAAGT, base positions 167–186) and an antisense primer specific to exon 9 (AATTCACAGTAATGTTCCCAAACAT; base positions 764–740) of the cDNA of murine CD45 (Thomas et al., 1987). cDNA was prepared by incubating 1 $\mu$g of poly $A^+$ RNA for 60 min at 37 °C with 200 units of MoMuLV reverse transcriptase in a 20-$\mu$l reaction volume containing 50 mM Tris-HCl (pH 8.3), 75 mM KCl, 3 mM $MgCl_2$, 5 mM DTT, 100 $\mu$g/ml BSA, 40 units RNasin, 500 $\mu$M dNTP, and 200 ng of antisense primer. A 5-$\mu$l aliquot was used directly for PCR amplification in a 50-$\mu$l reaction volume containing 50 mM KCl, 10 mM Tris-HCl (pH 9.3), 3 mM $MgCl_2$, 0.1% w/v gelatin, 500 $\mu$M dNTP, 400 ng of sense and antisense primers, and 2.5 units of Taq polymerase. PCR was performed in a DNA Thermal Cycler (Perkin-Elmer-Cetus, Inc.) for 24 cycles. Each cycle consisted of 40 s at 94 °C for denaturation, 15 s at 55 °C for annealing, and 30 s at 72 °C for elongation. The first cycle was preceded by a 5-min incubation at 94 °C and the last cycle followed by a 4-min incubation at 72 °C.

### Cytological Analyses

Cells were cytocentrifuged onto a microscope slide and allowed to air dry overnight. The cells were then incubated with either rat anti-B220 (monoclonal 14.8) or rat anti-MAC-1 (Boehringer Mannheim). Goat antirat immunoglobulin-horseradish peroxidase (Boehringer Mannheim) was used in a secondary incubation for detection. The presence of $\alpha$-naphthyl acetate esterase was determined by cytochemical staining (Yam et al., 1971) with a Sigma research kit. Nonspecific phagocytosis of latex beads was assayed by the method of Raschke et al. (1978).

### Antigen Presentation

Assays for antigen presentation were performed in a manner similar to that described by Marrack et al. (1989). Briefly, the cell lines to be assayed for antigen presentation were titrated into 200-$\mu$l microcultures containing $10^5$ cells of either the T-cell hybridomas DO-11.10/54.4 or 3Q023-24.4, both of which produce IL-2 in response to the presentation of chicken ovalbumin (cOVA) in the context of I-$A^d$ or I-$A^q$, respectively. These assays were carried out in RPMI 1640 supplemented with 10% fetal calf serum, $5\times10^{-5}$ M 2-mercaptoethanol and, where required, cOVA at 1 mg/ml. After 24 hr, incubation supernatants from these cultures were assayed for IL-2 using CTLL-2, an IL-2-dependent cytotoxic T-cell line. Twofold serial dilutions of supernatants were added to $5\times10^3$ CTLL-2 cells in 100-$\mu$l microcultures and incubated for 48 hr at 37 °C. MTT (Sigma), a substrate for production of a colored product indicative of cell survival (Mosmann, 1983), was added at 0.5 mg/ml and the cultures incubated for an additional 4 hr at 37 °C. Acid-isopropanol (40-mM HCl) was then added to dissolve the MTT formazan reaction product. The optical density of each well was quantitated by an ELISA reader at a wavelength of 540 nm. The specific activity of IL-2 in the supernatants was determined by comparison to a standard curve produced through the use of purified recombinant IL-2 (Cetus Inc.).

### Flow Cytometry

Cells were stained in PBS, 2% FCS with either FITC-conjugated monoclonal antibody AMS-32.1 (antimouse I-$A^d$) (Phar Mingen) or FITC-conjugated mouse $IgG_{2b}$, K (Phar Mingen) as an isotype-matched control. Cells were then fixed in PBS, 2% FCS, 0.5% formaldehyde, and stored at 4 °C until analysis. Flow cytometry was performed using an Ortho Diagnostics Cytofluorograph 50-H.

### ACKNOWLEDGMENTS

This work was supported by Public Health Service grants CA45360 (RCS) and GM35774 (WJE) and by a grant from the Elsa U. Pardee Foundation (RCS). We are indebted to Alfred Ayala for the cytokine assays. The authors thank Donna Paulnock for helpful discussions, and Susan Conrad and Michele Fluck for thoughtful comments on this manuscript.

*(Received October 8, 1991)*

*(Accepted November 22, 1991)*

### REFERENCES

Aviv H., and Leder P. (1975). Purification of biologically active globulin messenger RNA by chromatography on oligothymidylic acid-cellulose. Proc. Natl. Acad. Sci. USA **69:** 1408–1412.

Ayala A., Perrin M.M., Meldrum D.R., Eretel W., and Chaudry I.H. (1990a). Hemorrhage induces an increase in serum TNF which is not associated with elevated levels of endotoxin. Cytokin **3:** 170–175.

Ayala A., Perrin M.M., Wagner M.A., and Chaudry I.H. (1990b). Enhanced susceptibility to sepsis following simple hemorrhage: Depression of Fc and C3b receptor-mediated phagocytosis. Arch. Surg. **125:** 70–75.

Borzillo G.V., Ashmun R.A., and Sherr C.J. (1990). Macrophage lineage switching of murine early pre-B lymphoid cells expressing transduced *fms* genes. Mol. Cell. Biol. **10:** 2703–2714.

Boyd A.W., and Schrader J.W. (1982). Derivation of macrophage-like lines from the pre-B lymphoma ABLS 8.1 using 5-azacytidine. Nature **297:** 691–693.

Chang H.-L., Lefrancois L., Zaroukian M.H., and Esselman W.J. (1991). Developmental expression of CD45 alternate exons in murine T cells. Evidence of additional exon use. J. Immunol. **147:** 1687–1693.

Chang H.-L., Zaroukian M.H., and Esselman W.J. (1989). T200 alternate exon use in murine lymphoid cells determined by reverse transcription-polymerase chain reaction. J. Immunol. **143:** 315–321.

Chen S.-C., Redenius D., and Schwartz R.C. (1991). Tumorigenesis of a v-Ha-*ras*-expressing pre-B cell line selects for c-*myc* activation. Biochem. Biophys. Res. Comm. **178:** 1343–1350.

Chesnut R.W., Colon S.M., and Grey H.M. (1982). Antigen presentation by normal B cells, B cell tumors and macrophages: Functional and biochemical comparison. J. Immunol. **128:** 1764–1768.

Coffman R.L., and Weissman I.L. (1981). A monoclonal antibody that recognizes B cells and B cell precursors in mice. J. Exp. Med. **153:** 269–279.

Davidson W.F., Pierce J.H., Rudikoff S., and Morse H.C. (1988). Relationships between B cell and myeloid differentiation. J. Exp. Med. **168:** 389-407.

Donner L., Fedele L.A., Garon C.F., Anderson S.J., and Sherr C.J. (1982). McDonough feline sarcoma virus: Characterization of the molecularly cloned provirus and its feline oncogene (v-*fms*). J. Virol. **41:** 489–500.

Ellis R.W., De Feo D., Maryak J.M., Young H.A., Shih T.Y., Chang E.H., Lowy D.R., and Scolnick E.M. (1980). Dual evolutionary origin for the rat genomic genetic sequences of Harvey murine sarcoma virus. J. Virol. **36:** 408–420.

Fort P., Marty L., Piechaczyk M., El Salrouty S., Dani C., Jeanteur J., and Blanchard J.M. (1985). Various rat adult tissues express only one major mRNA species from the glyceraldehyde-3-phosphate dehydrogenase multigenic family. Nucl. Acids Res. **13:** 1431–1442.

Gonda T.J., and Metcalf D. (1984). Expression of *myb, myc* and *fos* proto-oncogenes during the differentiation of murine myeloid cells. Nature **310:** 249–251.

Greaves M.F., Chan L.C., Furley A.J.W., Watt S.M., and Molgard H.W. (1986). Lineage promiscuity in hemapoietic differentiation and leukemia. Blood **67:** 1–11.

Hanecak R., Zovich D.C., Pattengale P.K., and Fan H. (1989). Differentiation in vitro of a leukemia virus-induced B-cell lymphoma into macrophages. Mol. Cell. Biol. **9:** 2264–2268.

Holmes K.L., Pierce J.H., Davidson W.F., and Morse H.C. (1986). Murine hematopoetic cells with pre-B or pre-B/myeloid characteristics are generated by *in vitro* transformation with retroviruses containing *fes, ras, abl,* and *src* oncogenes. J. Exp. Med. **164:** 443–457.

Hultner L., Szots H., Welle M., Van Snick J., Moeller J., and Dormer P. (1989). Mouse bone marrow-derived interleukin 3-dependent mast cells and autonomous sublines produce interleukin 6. Immunology **67:** 408–413.

Kemp D.J., Harris A.W., and Adams J.M. (1980). Transcripts of the immunoglobulin C$\mu$ gene vary in structure and splicing during lymphoid development. Proc. Natl. Acad. Sci. USA **77:** 7400–7404.

Klinken S.P., Alexander W.S., and Adams J.M. (1988). Hemapoietic lineage switch: v-*raf* oncogene converts E$\mu$-*myc* transgenic B cells into macrophages. Cell **53:** 857–867.

Marrack P.; McCormack J., and Kappler J. (1989). Presentation of antigen, foreign major histocompatibility complex proteins and self by thymus cortical epithelium. Nature **338:** 503–505.

McCulloch E.A. (1983). Stem cells in normal and leukemic hemopoiesis. Blood **62:** 1–13.

Mosmann T. (1983). Rapid colorimetric assay for cellular growth and survival: application to proliferation and cytotoxicity assays. J. Immunol. Meth. **65:** 55–63.

Parnes J.R., Velan B., Felsenfeld A., Ramanathan L., Ferrini U., Appella E., and Seidman J.G. (1981). Mouse $\beta_2$-microglobulin cDNA clones: A screening procedure for cDNA clones corresponding to rare mRNAs. Proc. Natl. Acad. Sci. USA **78:** 2253–2257.

Ralph S.J., Thomas M.L., Morton C.C., and Trowbridge I.S. (1987). Structural variants of human T200 glycoprotein (leukocyte common antigen). EMBO J. **6:** 1251–1257.

Raschke W.G., Baird S., Ralph P., and Nakoinz I. (1978). Functional macrophage cell lines transformed by Abelson leukemia virus. Cell **15:** 261–267.

Rave N., Ckvenjakou R., and Blodtker H. (1979). Identification of procollagen mRNAs transferred to DBM paper from formaldehyde agarose gels. Nucl. Acids Res. **6:** 3559–3567.

Rigby P.W., Dieckmann M., Rhodes C., and Berg P. (1979). Labeling deoxyribonucleic acid to high specific activity *in vitro* by nick translation with DNA polymerase I. J. Mol. Biol. **113:** 237–251.

Rogers J., Early P., Carter C., Calame K., Bond M., Hood L., and Wall R. (1980). Two mRNAs with different 3' ends encode membrane-bound and secreted forms of immunoglobulin $\mu$ chain. Cell **20:** 303–312.

Saga Y., Tung J.S., Shen F.W., and Boyse E.A. (1987). Alternative use of 5' exons in the specification of Ly-5 isoforms distinguishing hematopoietic cell lineages. Proc. Natl. Acad. Sci. USA **84:** 5364–5368.

Schwartz R.C., Sonenshein G.E., Bothwell A., and Gefter M.L. (1981). Multiple expression of Ig $\lambda$-chain encoding RNA

species in murine plasmacytoma cells. J. Immunol. **126:** 2104–2108.

Schwartz R.C., Stanton L.W., Marcu K.B., and Witts O.N. (1986a). An *in vitro* model for tumor progresesion in murine lymphoid cells. Curr. Top. Microbiol. Immunol. **132:** 75–80.

Schwartz R.C., Stanton L.W., Riley S.C., Marcu K.B., and Witte O.N. (1986b). Synergism of v-*myc* and v-Ha-*ras* in the *in vitro* neoplastic progression of murine lymphoid cells. Mol. Cell. Biol. **6:** 3221–3231.

Seidman J.G., and Leder P. (1978). The arrangement and rearrangement of antibody genes. Nature **276:** 790–795.

Sheng-Ong G.L.C., Holmes K.L., and Morse H.C. (1987). Phorbol ester-induced growth arrest of murine myelomonocytic leukemic cells with virus disrupted *myb* locus is not accompanied by decreased *myc* and *myb* expression. Proc. Natl. Acad. Sci. USA **84:** 199–203.

Silver J., and Kozak C. (1986). Common proviral integration region on mouse chromosome 7 in lymphomas and myelogenous leukemias induced by Friend murine leukemia virus. J. Virol. **57:** 526–533.

Southern E.M. (1975). Detection of specific sequences among DNA fragments separated by gel electrophoresis. J. Mol. Biol. **98:** 502–517.

Springer T., Galfre G., Secher D.S., and Milstein C. (1979). Mac-1: A macrophage differentiation antigen identified by a monoclonal antibody. Eur. J. Immunol. **9:** 301–306.

Stanton L.W., Fahrlander P.D., Tesser P.M., and Marcu K.B. (1984). Nucleotide sequence comparison of normal and translocated murine c-*myc* genes. Nature **310:** 423–425.

Streuli M., Hall L.R., Saga Y., Schlossman S.F., and Saito H. (1987). Differential usage of three exons generates at least five different mRNAs encoding human leukocyte common antigens. J. Exp. Med. **166:** 1548–1566.

Thomas M.L., Reynolds P.J., Chain A., Ben-Neriah Y., and Trowbridge I.S. (1987). B-cell variant of mouse T200 (Ly-5): Evidence for alternative mRNA splicing. Proc. Natl. Acad. Sci. USA **84:** 5360–5363.

Thomas P. (1980). Hybridization of denatured RNA and small DNA fragments transferred to nitrocellulose. Proc. Natl. Acad. Sci. USA **77:** 5201–5205.

Walker E., Warner N.L., Chesnut R., Kappler J., and Marrack P. (1982). Antigen-specific, I region-restricted interactions *in vitro* between tumor cell lines and T cell hybridomas. J. Immunol. **128:** 2164–2169.

White J., Haskins K.M., Marrack P., and Kappler J. (1983). Use of I region-restricted, antigen-specific T cell hybridomas to produce idiotypically specific anti-receptor antibodies. J. Immunol. **130:** 1033–1037.

Whitlock C.A., Ziegler S.F., Treiman L.J., Stafford J.I., and Witte O.N. (1983). Differentiation of cloned populations of immature B cells after transformation with Abelson murine leukemia virus. Cell **32:** 903–911.

Yam L.T., Li C.Y., and Crosby W.H. (1971). Cytochemical identification of monocytes and granulocytes. Am. J. Clin. Path. **55:** 283–290.

